# Radioembolization Versus Bland Embolization for Hepatic Metastases from Small Intestinal Neuroendocrine Tumors: Short-Term Results of a Randomized Clinical Trial

**DOI:** 10.1007/s00268-017-4324-9

**Published:** 2017-11-22

**Authors:** Anna-Karin Elf, Mats Andersson, Olof Henrikson, Oscar Jalnefjord, Maria Ljungberg, Johanna Svensson, Bo Wängberg, Viktor Johanson

**Affiliations:** 1 Department of Surgery, Sahlgrenska University Hospital and Sahlgrenska Academy, University of Gothenburg, Gothenburg, Sweden; 2 Department of Radiology, Sahlgrenska University Hospital and Sahlgrenska Academy, University of Gothenburg, Gothenburg, Sweden; 3 Department of Radiation Physics, Sahlgrenska University Hospital and Sahlgrenska Academy, University of Gothenburg, Gothenburg, Sweden; 4 Department of Oncology, Sahlgrenska University Hospital and Sahlgrenska Academy, University of Gothenburg, Gothenburg, Sweden

## Abstract

**Background:**

Radioembolization (RE) with intra-arterial administration of ^90^Y microspheres is a promising technique for the treatment of liver metastases from small intestinal neuroendocrine tumors (SI-NET) not amenable to surgery or local ablation. However, studies comparing RE to other loco-regional therapies are lacking. The aim of this randomized study was to compare the therapeutic response and safety after RE and bland hepatic arterial embolization (HAE), and to investigate early therapy-induced changes with diffusion-weighted MRI (DWI-MRI).

**Methods:**

Eleven patients were included in a prospective randomized controlled pilot study, six assigned to RE and five to HAE. Response according to RECIST 1.1 using MRI or CT at 3 and 6 months post-treatment was recorded as well as changes in DWI-MRI parameters after 1 month. Data on biochemical tumor response, toxicity, and side effects were also collected.

**Results:**

Three months after treatment, all patients in the HAE group showed partial response according to RECIST while none in the RE group did (*p* = 0.0022). After 6 months, the response rates were 4/5 (80%) and 2/6 (33%) in the HAE and RE groups, respectively (NS). DWI-MRI metrics could not predict RECIST response, but lower pretreatment ADC_(120–800)_ and larger ADC_(0–800)_ increase at 1 month were related to larger decrease in tumor diameter when all tumors were counted.

**Conclusion:**

HAE resulted in significantly higher RECIST response after 3 months, but no difference compared to RE remained after 6 months. These preliminary findings indicate that HAE remains a safe option for the treatment of liver metastases from SI-NET, and further studies are needed to establish the role of RE and the predictive value of MR-DWI.

**Electronic supplementary material:**

The online version of this article (10.1007/s00268-017-4324-9) contains supplementary material, which is available to authorized users.

## Introduction

The primary tumors of small intestinal neuroendocrine tumors (SI-NETs) can almost always be radically resected by surgery, often together with the regional lymph node metastases. Hepatic metastases are common, and surgical resection is suitable only in a minority of cases calling for other loco-regional treatment alternatives [1]. Local ablative therapies using radiofrequency or microwaves are an option if the metastases are few, small, and suitably localized. In other cases, loco-regional endovascular therapies, such as hepatic arterial embolization (HAE) or chemoembolization (TACE), are considered. The mainstay for treating widespread liver metastases at our unit has been HAE with polyvinyl alcohol (PVA) particles [2]. However, the treatment is often associated with side effects due to liver ischemia, and recurrence may occur. Recently, radioembolization (RE), involving transcatheter arterial delivery of 20–60 µm microspheres containing ^90^Y radioisotope into the tumor microvasculature, has emerged as a promising tool in the management of hepatic metastases from NET. RE is considered to have less acute and subacute toxicity than HAE, as it avoids liver ischemia. However, studies documenting the safety and efficacy of RE largely represent retrospective cohort series [3–9], while prospective studies comparing RE to other loco-regional therapies such as HAE are lacking.

Currently accepted guidelines for assessing treatment response in solid tumors include response evaluation criteria in solid tumors (RECIST) [10, 11]. However, these morphological criteria may not be conclusive until several months following therapy. Earlier evaluation of response to therapy would allow alternative therapy sooner, with optimized management of the individual patient. Diffusion-weighted imaging (DWI) is a functional MR imaging method that provides insight into the tumor microenvironment and can potentially be used as an early surrogate biomarker for tumor response [12, 13].

Here, we present the preliminary results of a randomized phase II study where the primary end point was to compare the treatment response, according to RECIST, of hepatic metastases at 3 months after RE or HAE. Secondary aims were to study the radiological response at 6 months, the biochemical response, and toxicity, and to evaluate the usefulness of early changes in DWI parameters in predicting later treatment response.

## Materials and methods

### Patients

Inclusion criteria were multiple SI-NET liver metastases, grade 1 or 2, not accessible to curative resection or ablation, and elevated serum chromogranin A (CgA) and/or 24 h urinary 5-HIAA excretion (dU-5HIAA). All patients were under somatostatin analogue (SSA) treatment, and primary surgery with removal of all extrahepatic tumors had been performed in all patients.

Exclusion criteria were remaining extrahepatic metastases, previous loco-regional or systemic anti-tumoral treatment (except SSA), impaired liver function or tumor volume exceeding 50% of total liver volume.

Before treatments, all patients but one were investigated with DWI-MRI. The remaining patient could not undergo MRI due to cardiac pacemaker and was evaluated with CT. DWI-MRI was repeated 1 month after treatment followed by response evaluation with MRI or CT according to RECIST 1.1 at 3 and 6 months.

The study was approved by the Ethical Review Board of Gothenburg University.

### RE and HAE procedures

Radioembolization was performed with bilobar infusion in a standard manner in all patients [9]. Protective coil embolization was used when necessary to prevent non-target embolization. The administered activity of ^90^Y resin microspheres (SIR-spheres™) was calculated using the partition model [14].

HAE was performed by infusion of PVA particles (45–150 μm) into the right or left hepatic artery until stasis was achieved. The right liver lobe was treated first, embolizing the remaining left lobe at a second session, about 6 weeks later. No other anti-tumoral therapy was started during the study period.

### Magnetic resonance imaging

The MRI measurements were performed on a Gyroscan Achieva dStream 3 T Release 5.1.7 (Philips Medical Systems, Eindhoven, the Netherlands). The examination included T2W and T1W scans, and a DWI scan with multiple *b*-values (0, 120, 350, 575, 800 s/mm^2^). At baseline and at the 3 and 6 months follow-up examinations, a T1-weighted dynamic contrast-enhanced sequence in the late arterial and portal venous phases was added to the protocol (for details see Electronic Supplementary Material).

### Image analysis

Up to five liver metastases, with lesion diameters larger than 1 cm, were analyzed per patient. Multiple free-hand regions of interest (ROIs) were drawn to encompass the whole volume of the metastases on DWI-MRI images. The relative ADC values at 1 month, compared to pretreatment baseline values, were calculated. As a control, a circular ROI of minimum 120 mm^2^ was placed on normal-appearing splenic parenchyma (for details see Electronic Supplementary Material).

### Assessment of therapeutic response

Response evaluation was performed by MRI or CT at 3 and 6 months after treatment, according to the RECIST 1.1 guidelines [11]. For every patient, two target lesions, which appeared best measurable and representative of the patient’s overall tumor load at the baseline MRI/CT, were chosen in accordance with RECIST criteria using the “Tumor Tracking” module at the IntelliSpace-Portal workstation.

Biochemical tumor markers CgA in serum and dU-5HIAA were measured at 3 and 6 months after treatment.

Therapeutic response at 3 and 6 months after treatment was also determined on a lesion-by-lesion basis by evaluating changes in size of a total of 39 tumors. The measurements of the lesion diameters (LD = longest diameter) were averaged across all MR sequences. The relative tumor size at different times after treatment was calculated by comparing it with pretreatment baseline values (for details see Electronic Supplementary Material).

### Toxicity

Toxicity was assessed weekly during the first month after treatment by measuring hemoglobin, white blood count, and platelets. At the same time points, liver-specific toxicity was assessed by measuring ASAT, ALAT, ALP, and bilirubin. Blood sampling was repeated at 3 and 6 months after treatments.

### Statistical analysis

Response rates according to RECIST1.1 were estimated with binomial proportions and compared by Fisher’s exact test using the MedCalc statistics package (MedCalc Software, Ostend, Belgium). Linear mixed models were used to test for difference between changes in ADC parameters and for linear correlation between ADC parameters and change in size following treatment using MATLAB 2016b (MathWorks Inc, Natick, MA, USA). Mann–Whitney *U* test or Wilcoxon signed-rank test were used to compare differences in continuous variables. A *p* value of <0.05 was considered statistically significant.

## Results

Between January 2014 and September 2016, 11 patients with irresectable liver metastases from SI-NET, not suitable for local ablative treatments, were randomly assigned to RE (*n* = 6) or HAE (*n* = 5). Median age at enrollment was 67 years, and three patients were men (Table 1). All patients had > 5 metastases, and a total of 36 lesions, which could be consistently assessed throughout all MRI examinations, were analyzed on a lesion-by-lesion basis. A median activity of 1.4 (range 1.1–1.8) GBq of ^90^Y was given to patients receiving RE. The first radiological response evaluation, DWI-MRI, was performed after a median time of 32 days (range 22–55 days) after RE or the first HAE treatment (the right lobe). The 2nd and 3rd evaluations were performed after a median time of 90 and 195 days, respectively (range 84–118 and 166–251 days), after treatment. However, one of the patients, with a pacemaker, was assessed with CT only, and another patient treated with HAE failed to undergo DWI-MRI at the 1-month time point.

**Table 1:** Baseline data: Baseline clinical and tumor characteristics of the study population randomized to RE or HAE

	All patients (*n* = 11)	Patients receiving RE (*n* = 6)	Patients receiving HAE (*n* = 5)	*p* value
Age, years, median (range)	67 (40–79)	66.5 (40–79)	67 (51–79)	0.65*
Male sex	3	2	1	1.00**
Number of lesions analyzed, median (range)	4 (1–5)	5 (2–5)	3 (1–5)	0.11*
Median LD, mm (range)	20.3 (13–55)	20.3 (13–50)	19.9 (13–55)	0.92*
Median sum of LD of metastases analyzed, mm (range)	77 (30–170)	89 (35–170)	74 (30–170)	0.27*
Median baseline ADC_(120–800)_, 10^−3^mm^2^/s (range)	0.73 (0.5–1.3)	0.78 (0.5–1.3)	0.68 (0.5–1.0)	0.15*
Primary tumor, grade 1 (Ki-67 < 2%) (*n*)	7	5	2	0.24**
Primary tumor, grade 2 (Ki-67 3–20%) (*n*)	4	1	3	0.24**
Median dU-5HIAA (µmol/24 h) (range)	110 (21–270)	97 (54–130)	110 (21–270)	0.93*
Median CgA (µg/L) (range)	231 (81–1890)	162 (81–470)	384 (115–1890)	0.36*

* Mann–Whitney *U* test

** Fisher’s exact test

### RECIST response

**Fig. 1:**
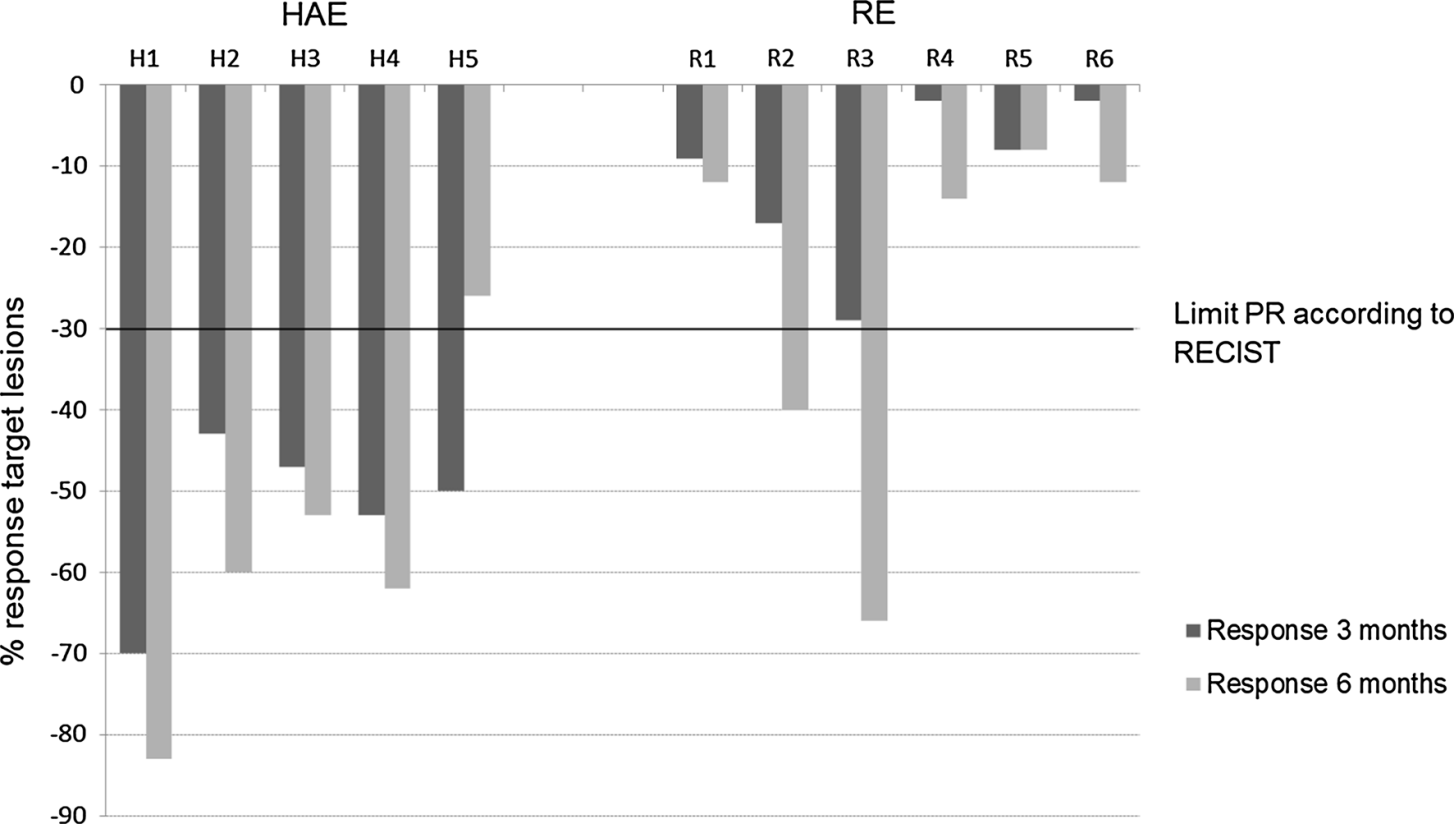
RECIST response in target lesions in the treated liver at 3 months (dark gray staples) and 6 months (light gray staples) post-treatment. Solid black line indicates threshold for partial response (PR). At 3 months, no responders were seen in the RE group while all patients in the HAE group showed PR (*p* = 0.0022)

At 3 months after treatment, no responders were seen in the RE group while all patients in the HAE group showed partial response (PR) (*p* = 0.0022). After 6 months, two patients in the RE group showed PR while one patient in the HAE group had progressed compared to nadir at 3 months, resulting in no remaining significant difference between the groups (Fisher’s exact test, *p* = 0.24) (Fig. 1).

### Biochemical response

**Fig. 2:**
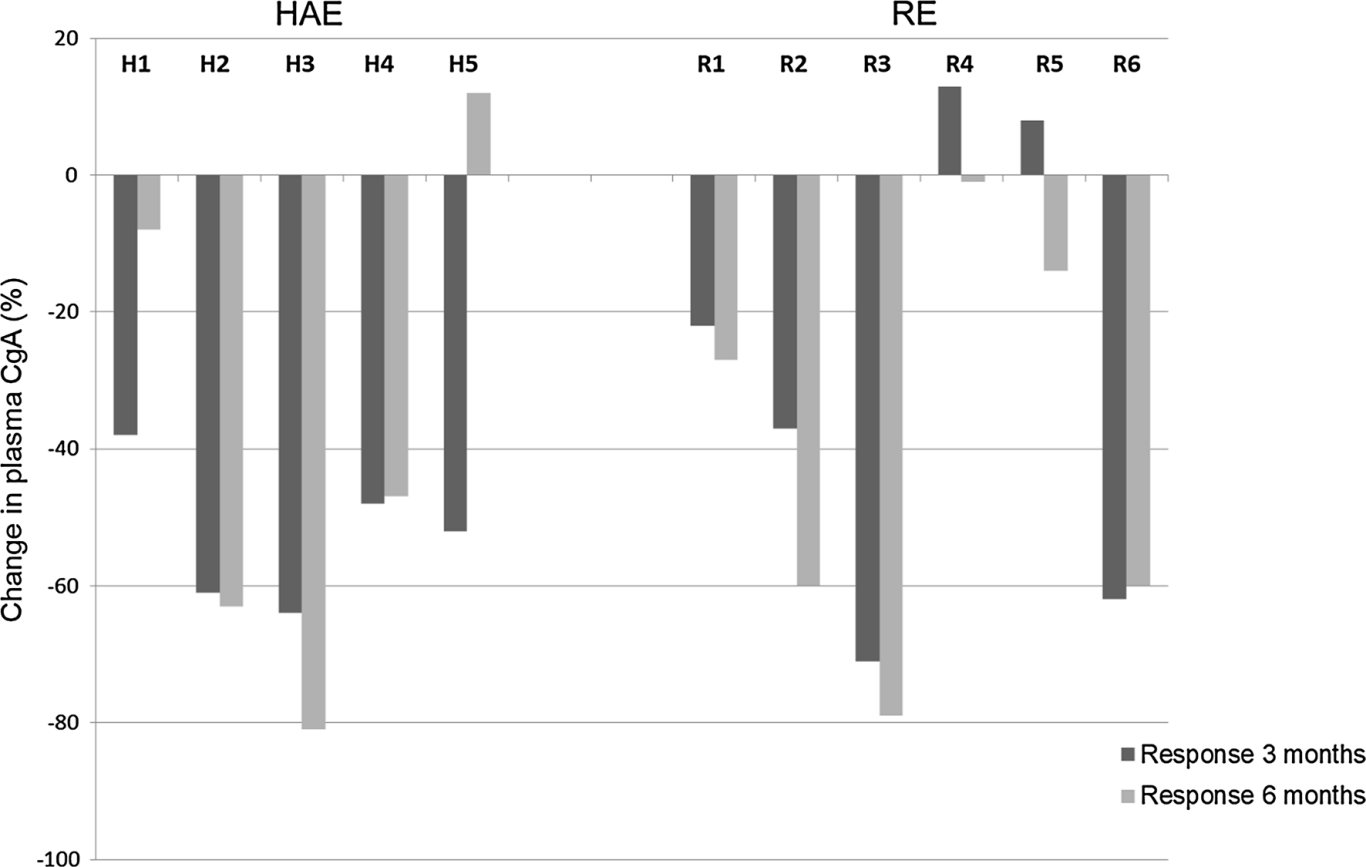
Relative change in CgA levels compared to baseline values, at 3 months (dark gray staples) and at 6 months (light gray staples) after treatment with HAE or RE. The median decrease in CgA levels at 3 months was 52% in the HAE group and 29% in the RE group, and 47 and 44%, respectively, at 6 months (NS, *p* = 0.42 and 0.66, respectively)

**Fig. 3:**
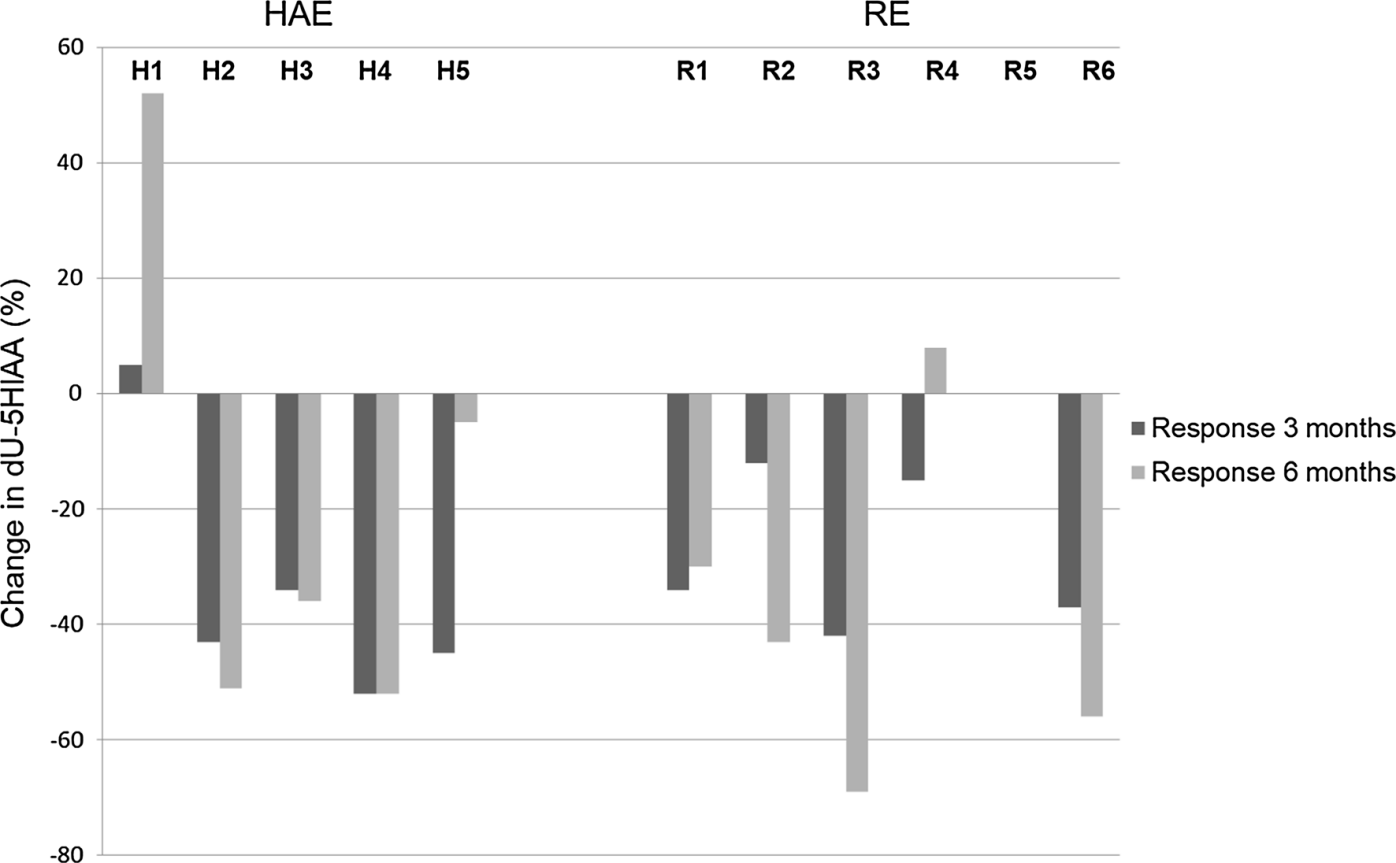
Relative change in dU-5HIAA compared to baseline values, at 3 months (dark gray staples) and at 6 months (light gray staples) after treatment with HAE or RE. The median decrease in dU-5HIAA at 3 months was 43% in the HAE group and 25% in the RE group, and 36 and 43%, respectively, at 6 months (NS, *p* > 0.05). Patient R5 showed no change in dU-5HIAA values at all time points, hence no visible bars in the figure

Biochemical markers, CgA and 5HIAA, decreased in most patients after 3 and 6 months. The data were rather divergent, and there were no statistically significant differences between the treatment groups. The median reduction in CgA level after 3 months was 52% in the HAE group and 29% in the RE group. Corresponding values after 6 months were 47 and 44%, respectively (Fig. 2). The median reduction in 5HIAA excretion after 3 months was 43% in the HAE group and 25% in the RE group. Corresponding values after 6 months were 36 and 43%, respectively (Fig. 3).

### Tumor size response and correlation with DWI assessment

**Fig. 4:**
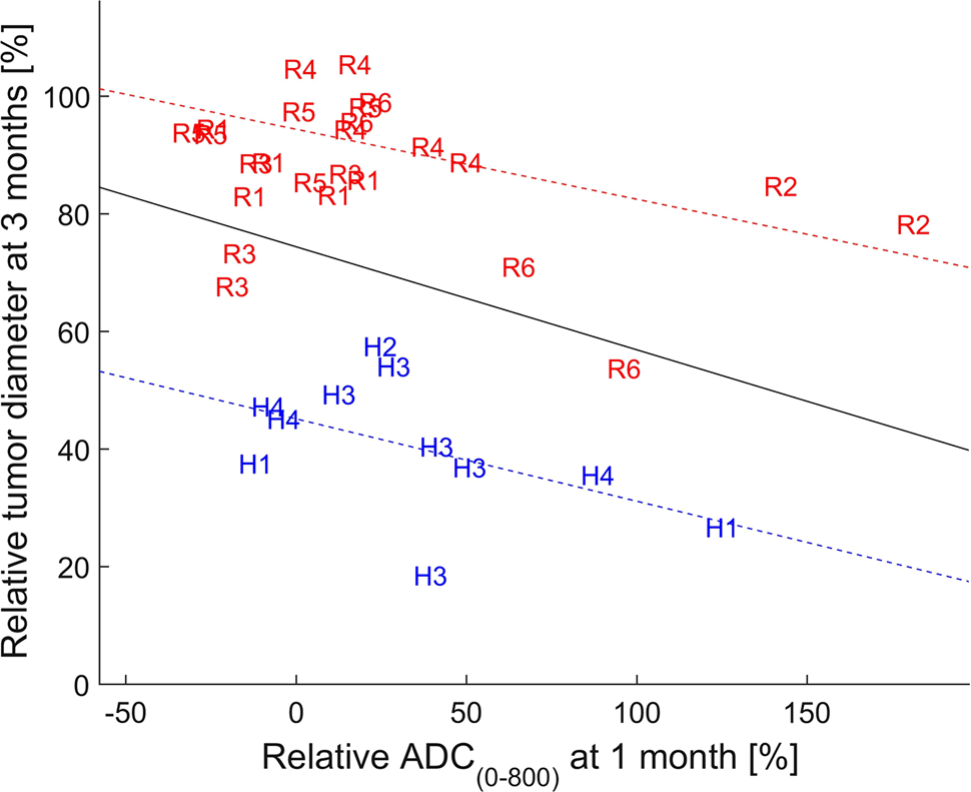
Correlation between ADC_(0–800)_ at 1 month and decrease in LD at 3 months (*k* = −0.2, *p* < 0.05) in individual tumors in patients treated with RE (R) and HAE (H), respectively. Values are given as percent of pretreatment value. The blue, red, and black lines show the regression line for HAE patients, RE patients, and all patients, respectively

**Fig. 5:**
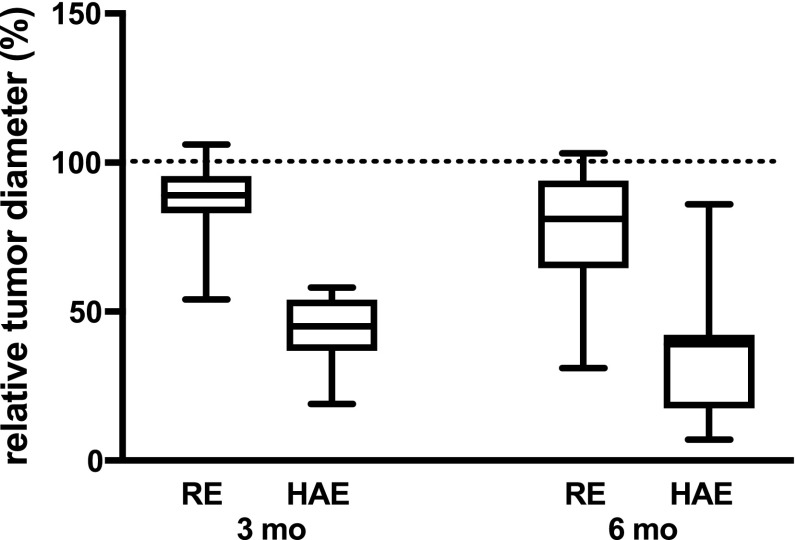
Relative individual tumor diameters after treatment. At 3 months after treatment, the relative (compared to baseline) median tumor diameter in measured lesions was significantly smaller after HAE, 45% (range 19–58%), compared to RE, 89% (range 54–106%), (*p* < 0.001). The difference remained after 6 months, when the relative tumor diameter was 39% (range 7–86%) after HAE and 81% (range 31–103%) after RE (*p* < 0.001)

The median pretreatment LD was 20 mm (range 13–55 mm) when evaluating all 36 tumors. In the 10 patients examined with MRI, pretreatment ADC_(120–800)_ values were significantly negatively correlated with treatment response (decreasing LD) at 6 months (*k* = 45, *p* < 0.01). The mean increase in ADC_(120–800)_ at 1 month was 40% after HAE and 11% after RE, although the difference between treatments was not statistically significant (*p* = 0.08). However, there was a statistically significant correlation between the relative increase in the ADC_(0–800)_ values at 1 month and treatment response (decreasing LD) at 3 months (*k* = −0.2, *p* < 0.05) (Fig. 4). After 3 and 6 months, the relative (compared to baseline) median LD was significantly smaller in metastases treated with HAE than in patients treated with RE, 45 versus 89% (*p* < 0.001) and 39 versus 81% (*p* < 0.001), respectively (Fig. 5).

No difference in the median ADC_(0–800)_ or ADC_(120–800)_ values measured in the spleen before and after treatment was found (data not shown).

### Toxicity

Overall toxicity from both treatments was low, and blood cell count at 6 months did not differ from baseline. Liver-specific evaluation showed that RE patients had slightly increased median levels of alkaline phosphatase compared to HAE patients at 6 months (2.8 µkat/L, range 1.6–15.5 vs. 1.45 µkat/L, range 1.0–2.1, *p* < 0.05), but no significant differences in liver enzymes or bilirubin values were found.

One patient in the HAE group developed post-treatment cholecystitis, which caused a prolonged hospital stay of 10 days. Median hospital stay after HAE was 4 days (range 4–10 days), which was significantly (*p* = 0.024) longer than after RE (median 2 days, range 2–6 days).

## Discussion

For SI-NET patients with metastases that cannot be resected by surgery, other treatment options are needed. Systemic radiotherapy with radiolabeled somatostatin analogues (PRRT) has recently proved to be superior to treatment with somatostatin analogues alone [15]. But accumulated radiotoxicity is a concern, and repeated full-dose PRRT can rarely be performed. When the metastatic burden is limited to the liver, liver-directed therapy is preferable. At least HAE can be performed repeatedly, and neither HAE nor RE prevents future use of PRRT, if needed [16].

This is the first randomized prospective study that compares HAE and RE for the treatment of hepatic SI-NET metastases. Response evaluation according to RECIST utilizes the longest lesion diameter (LD) and specified threshold diameter changes. However, the appearance and size of liver metastases from NET may vary between MR sequences and phases of contrast administration [17]. It is currently somewhat unclear which sequence is the best for measuring these lesions [18]. Peri-tumoral edema on T2W imaging and peri-tumoral enhancement on dynamic contrast imaging may cause overestimation of the diameter measured. We hypothesized that averaging LD across all imaging sequences at one time point would compensate for these differences, optimizing the conditions for accurate response evaluation.

In this study, the RECIST response rate after radioembolization for metastatic NET was 33% at 6 months, which compares well with other studies that have shown a response rate between 22 and 63% [4–6, 19–22]. We noted that compared to HAE, the response after RE was delayed and that the target lesions continued to decrease in size from 3 to 6 months post-treatment. At 6 months, no significant difference in the RECIST response remained. A similar trend in the response pattern of the biochemical tumor markers CgA and 5HIAA was noted. Such a delayed treatment response has also been observed by Fidelman et al. [23] who reported a median time to maximum response of 11 months in a study using ^90^Y glass microspheres for the treatment of metastatic NET.

ADC_(120–800)_ primarily reflects diffusion, and both animal models and clinical studies have shown that lower ADC values in tumors indicate high viability [12] and a relative lack of necrosis, facilitating the intratumoral delivery of therapeutic agents [24, 25]. The finding that lower pretreatment ADC_(120–800)_ was related to improved response (larger decrease of LD at 6 months) represents a well-known association between pretreatment ADC and response that has been reported for NET as well as for other tumor entities [26–28].

While HAE resulted in a significant increase in ADC_(120–800)_ at 1 month, RE did not. The reason for this is not clear, although the radiotoxic effect certainly is delayed compared to the more immediate ischemic effect of bland embolization. Another study of RE found no early ADC increase in responding colorectal liver metastases, which they associated with cellular edema [29]. Signs of an inflammatory response with peri-tumoral edema after RE have also been described on CT imaging [30]. However, it cannot be ruled out that the lack of a significant increase in diffusion at 1 month after RE was due to inadequate anti-tumor effect or suboptimal timing of the DWI measurement.

DWI analysis at 1 month after treatment could not predict the RECIST response in an individual patient in this small study. A larger increase in ADC_(0–800)_ was, however, related to improved response (larger decrease of LD at 3 months) when analyzing all measured lesions. Since ADC_0–800_, as opposed to ADC_120–800_, is affected by both diffusion and pseudodiffusion caused by microscopic circulation, this result may indicate that changes in microperfusion after embolization are involved in the therapeutic process. Although there are contradictory results in the literature [31], this finding is in accordance with the suggestion of Kukuk et al. [26] that there is an increase in microperfusion secondary to a decrease in interstitial fluid pressure (IFP) in responding NET metastases.

This study has several limitations. Being an interim analysis of a pilot study, the study population is small, which limits the conclusions that can be drawn. The effect of inter-observer variation in the ROI positioning was not investigated, but it has been shown that this variation is less important in whole-volume measurements, as in our study [32].

Strength of the study is the prospective randomized design. To our knowledge, this is the first randomized trial comparing outcome after RE versus HAE in patients with metastatic NET.

In conclusion, in this randomized pilot study comparing RE and HAE for liver metastatic SI-NET, all metastases decreased in size after 3 months, but the decrease was significantly larger in patients treated with HAE. However, no significant difference in RECIST response between groups remained after 6 months, suggesting a delayed anatomical response after treatment with RE. Although lower pretreatment ADC_(120–800)_ and larger increase in ADC_(0–800)_ at 1 month were related to improved response (decrease in tumor diameter), at 3 and 6 months respectively, no DWI-MRI threshold for the prediction of response according to RECIST could be defined. These preliminary findings indicate that HAE remains a safe option for treatment of liver metastases from SI-NET, and further studies are needed to establish the role of RE and the predictive value of DWI-MRI in these tumors.

## Electronic supplementary material

Below is the link to the electronic supplementary material.

Supplementary material 1 (DOCX 13 kb)
