# Supplementary material for: Radioembolization Versus Bland Embolization for Hepatic Metastases from Small Intestinal Neuroendocrine Tumors: Short-Term Results of a Randomized Clinical Trial
Source: World J Surg. 2017 Nov 22;42(2):506–13. doi: 10.1007/s00268-017-4324-9 (PMC5762793; doi:10.1007/s00268-017-4324-9)
Supplement: Supplementary file 1 — Supplementary material 1 (DOCX 13 kb) [file 268_2017_4324_MOESM1_ESM.docx]

**ELECTRONIC SUPPLEMENTAL MATERIAL**

**Under Materials and Methods section**

**Magnetic resonance imaging**

The MRI measurements were performed on a Gyroscan Achieva dStream 3 T Release 5.1.7 (Philips Medical Systems, Eindhoven, the Netherlands). The examination included a survey, a T2W TSE scan, a T1W mDIXON scan and a DWI-scan. The respiratory triggered DWI acquisition was performed with 5 b-values (0, 120, 350, 575, 800 s/mm^2^) and with (6, 3, 3, 6, 6) signal averages for each corresponding b-value. The in-plane resolution was 3×3 mm^2^ with a slice thickness of 6 mm with a slice-gap of 0.6 mm. TR and TE were set to shortest, resulting in a TR around 2600 ms and a TE of 54 ms. At baseline and at the 3 and 6 month follow-up examinations, a T1-weighted dynamic contrast enhanced sequence in the late arterial and portal venous phases was added to the protocol.

**Image analysis**

Diffusion-weighted MR images were reviewed on a dedicated post-processing work-station (IntelliSpace Portal, Philips, Eindhoven, the Netherlands). Up to five liver metastases with lesion diameters larger than 1 cm were analyzed per patient. One radiologist (MA) with more than 20 years’ experience in liver imaging drew multiple free-hand regions of interest (ROIs) to encompass the whole volume of the metastases on the b=800 s/mm^2^ images. Areas or slices obviously affected by motion artefacts or susceptibility artefacts, as well as lesions in the subdiaphragmatic region prone to artifacts were excluded. Each ROI was copied into the respective parameter map (ADC _(0-800)_, ADC _(120-800)_, ADC _(0-120)_), the mean apparent diffusion coefficient (ADC) of each ROI was recorded and an average ADC value was obtained for each lesion.

The percentage of ADC changes were calculated by using the formula (ADC _1 month_ – ADC _B_)/ ADC _B_ , where ADC _B_ was ADC pretreatment value and ADC _1 month_ was the respective ADC value on MRI one month after therapy. As a control, a circular ROI of minimum 120 mm^2^ was placed on normal-appearing splenic parenchyma.

**Assessment of therapeutic response**

Changes in tumor size after treatment were calculated by using the formula (LD _Follow-up_ ^–^ LD _B_)/ LD _B_, where LD _B_ was lesion size at baseline and LD _Follow-up_ was lesion size at the respective follow-up examination.
